# Supplementary material for: Differentiating wild from captive animals: an isotopic approach
Source: PeerJ. 2023 Nov 24;11:e16460. doi: 10.7717/peerj.16460 (PMC10680447; doi:10.7717/peerj.16460)
Supplement: Supplemental Information 2 — In “Summary Results” the equal sign (=) indicates the absence of significant differences in inferential tests. [file peerj-11-16460-s002.docx]

**Table S2.** Summary of studies using stable isotopes to differentiate between wild and captive animals organized by taxon group (fish, amphibian, reptile, bird, and mammal). In “Summary Results” the equal sign (=) indicates the absence of significant differences in inferential tests.

|  | **TAXON (SPECIE)** | **ISOTOPES ANALYZED** | **TISSUE** | **LOCAL (COUNTRY)** | **SUMMARY RESULT** | **REFERENCE** |
| --- | --- | --- | --- | --- | --- | --- |
|  | *Salmo salar* | *δ*^13^C, *δ*^15^N | Muscle | Canada | *δ*^13^C_wild_ < *δ*^13^C_captive_  *δ*^15^N_wild_ > *δ*^15^N_captive_ | (Dempson & Power, 2004) |
| **FISH** | *Sparus aurata* | *δ*^13^C, *δ*^15^N | Muscle | Italy;  France | *δ*^13^C_wild_ > *δ*^13^C_captive_  (Based on no overlaps between groups) | (Rojas et al., 2007) |
|  | *Dicentrarchus labrax* | *δ*^13^C, *δ*^15^N, *δ*^18^O | Muscle oil | England; Scotland; Greece | *δ*^13^C_wild_ > *δ*^13^C_captive_  *δ*^15^N_wild_ < *δ*^15^N_captive_  *δ*^18^O_wild_ = *δ*^18^O_Ncaptive_ | (Bell et al., 2007)^*^ |
|  | *Salmo salar* | *δ*^13^C, *δ*^15^N,  *δ*^18^O | Muscle | Ireland and Norway | *δ*^13^C_wild_ range < *δ*^13^C_cap-conventional_ range  *δ*^15^N_wild_ variation > *δ*^13^C_captive_ variation  *δ*^15^N_wild_ < *δ*^15^N_cap-organic_  *δ*^18^O_wild_ = *δ*^18^O_cap-conventional_ = *δ*^18^O_cap-organic_ | (Molkentin et al., 2007) |
|  | *Sparus aurata* | *δ*^13^C, *δ*^15^N | Muscle (red and white), liver and gills | Spain | *δ*^13^C_wild_ > *δ*^13^C_captive_  *δ*^15^N_wild_ < *δ*^15^N_captive_ | (Serrano, Blanes & Orero, 2007) |
|  | *Psetta maxima* | *δ*^13^C, *δ*^15^N | Muscle | Denmark, Spain, Netherlands | *δ*^13^C_wild_ > *δ*^13^C_captive_  *δ*^13^C_wild-Netherlands_ > *δ*^13^C_captive_  *δ*^15^N_wild-Netherlands_ > *δ*^15^N_wild-Denmark_ > *δ*^15^N_captive_ | (Busetto et al., 2008) |
|  | *Oncorhynchus tshawytscha; O. kisutch; Salmo salar* | *δ*^13^C, *δ*^15^N | Muscle | Pacific and Atlantic Ocean | 86 – 100% hits using different multivariate analyses: LDA, QDA, NN, PNN, and NNB | (Anderson, Hobbie & Smith, 2010) |
|  | *Dicentrarchus labrax* | *δ*^13^C, *δ*^15^N | Muscle | FAO zone 37.1 and 27 | *δ*^13^C_wild_ > *δ*^13^C_captive_  *δ*^15^N_wild_ > *δ*^15^N_captive_ | (Fasolato et al., 2010) |
|  | *Pseudoplatystoma fasciatum* | *δ*^13^C, *δ*^15^N | Muscle | Brazil | *δ*^13^C_wild_ < *δ*^13^C_captive_  *δ*^15^N_wild_ < *δ*^15^N_captive_ (rainy season) | (Sant’Ana, Ducatti & Ramires, 2010) |
|  | *Salmo salar; Oncorhynchus mykiss* | *δ*^13^C, *δ*^15^N | Muscle | Chile | *δ*^13^C_wild_ < *δ*^13^C_captive_  *δ*^15^N_wild_ < *δ*^15^N_captive_  93.9% hits in Discriminant Analysis | (Schröder & Garcia de Leaniz, 2011) |
|  | *Salmo trutta* | *δ*^13^C, *δ*^34^S | Scale | Poland | *δ*^13^C_wild_ *<δ*^13^C_captive_  *δ*^34^S_captive_ > *δ*^34^S_wild_  (Based on no overlaps between groups) | (Trembaczowski, 2011) |
|  | *Oncorhynchus nerka; O. kisutch; Salmo salar; S. trutta* | *δ*^13^C, *δ*^15^N | Muscle | United States, Ireland, Scotland, Norway, Germany | Bulk: *δ*^13^C_wild,_ *δ*^13^C_organic_ > *δ*^13^C_conventional_  *δ*^15^N_wild,_ *δ*^15^N_organic_ > *δ*^15^N_conventional_  Lipids: *δ*^13^C_organic_ > *δ*^13^C_wild,_ *δ*^13^C_conventional_  (Based on no overlaps between groups) | (Molkentin et al., 2015) |
|  | *Argyrosomus regius* | *δ*^13^C, *δ*^15^N | Muscle | Portugal | *δ*^13^C_wild_ > *δ*^13^C_captive_  *δ*^15^N_wild_ > *δ*^15^N_captive_ | (Chaguri et al., 2017) |
| **FISH** | *Dicentrarchus labrax* | *δ*^13^C, *δ*^15^N | Muscle | Europe | 91% hits in the Discriminant Analysis | (Farabegoli et al., 2018) |
|  | *Oncorhynchus gorbuscha,*  *Oncorhynchus nerka*  *Salmo salar* | *δ*^13^C, *δ*^15^N | Muscle | United States, Norway, Ireland | *δ*^13^C_bulk_: differences between all groups (except wild *vs.* Irish organic *S. salar*; wild *O. gorbuscha vs.* wild *O. nerka* salmon)  *δ*^15^N_bulk_: differences between wild and conventionally farmed  SCIA allowed more accurate results | (Wang et al., 2018) |
|  | *Lates calcarifer* | *δ*^13^C e *δ*^15^N | Muscle | Australia; Malaysia | *δ*^13^C_wild_ > *δ*^13^C_captive_  (except for Northern Territory – AU: no difference was found)  *δ*^15^N_wild_ > *δ*^15^N_captive_ | (Gopi et al., 2019) |
|  | *Arapaima spp.* | *δ*^13^C | Otolith | Brazil | *δ*^13^C_wild_ < *δ*^13^C_captive_  (Madeira, Solimões and Lower Amazon)  *δ*^13^C_wild_ > *δ*^13^C_captive_  (Central Amazon basin)  58% hits in the Discriminant Analysis | (Pereira et al., 2019) |
|  | *Anguilla anguilla* | *δ*^13^C, *δ*^15^N | Muscle | Italy, Denmark, Netherlands | Italy, Denmark, and Netherlands:  *δ*^13^C_wild-sea_ < *δ*^13^C_cap-int.-males_ < *δ*^13^C_wild-lagoon_ = *δ*^13^C_cap-ext_ = *δ*^13^C_cap-int-females_  *δ*^15^N_wild-sea_ = *δ*^15^N_cap-ext._ = *δ*^15^N_cap-int-female_ > *δ*^15^N_wild-lagoon_ = *δ*^15^N_cap-int-males_  Italy:  *δ*^13^C_wild-sea_ < *δ*^13^C_wild-lagoon_ = *δ*^13^C_cap-ext._  *δ*^15^N_wild-sea_ = *δ*^15^N_cap-ext._ > *δ*^15^N_wild-lagoon_ | (Vasconi et al., 2019) |
|  | *Oncorhynchus mykiss* | *δ*^13^C, *δ*^15^N | Muscle | Argentina | *δ*^13^C_wild_ < *δ*^13^C_captive_  *δ*^15^N_captive-farmC_ *> δ*^15^N_wild_ _=_ *δ*^15^N_captive-farmB_ > *δ*^15^N_captive-farmA_ | (Nabaes Jodar, Cussac & Becker, 2020) |
|  | *C. carpio; C. idella; H. molitrix; M. piceus* | *δ*^13^C, *δ*^15^N | Muscle, scale | China | *δ*^13^C_wild_ < *δ*^13^C_lake-farmed_ = *δ*^13^C_pond-farmed_  *δ*^15^N_wild_ < *δ*^15^N_pond farmed_  Discriminant model using isotopic and elemental data: 95-100% hits | (Liu et al., 2020) |
| **AMPHIBIAN** | *Hoplobatrachus rugulosus*,  *Fejervarya cancrivora; Limnonectes macrodon* | *δ*^13^C, *δ*^15^N, *δ*^18^O | Muscle, bone | Vietnam; Indonesia | Muscle: differences in *δ*^13^C, *δ*^15^N;  SD*δ*^15^N_wild_ > SD*δ*^15^N_farmed_  Bone: differences in *δ*^13^C and *δ*^18^O (Vietnam x Indonesia) | (Dittrich, Struck & Rödel, 2017) |
| **REPTILE** | *Shinisaurus crocodilurus* | *δ*^13^C, *δ*^15^N | Skin | Vietnam | *δ*^13^C_wild_ < *δ*^13^C_captive_; *δ*^15^N_wild_ < *δ*^15^N_captive_  Assignment test_wild x captive_: 100% hits | (van Schingen et al., 2016) |
|  | *Python reticulatus*;  *Python bivittatus* | *δ*^13^C, *δ*^15^N, *δ*^2^H | Skin | Vietnam; Indonesia | *P. bivitattus:*  *δ*^13^C_wild_ < *δ*^13^C_captive_; *δ*^15^N_wild_ > *δ*^15^N_captive;_ *δ*^2^H_wild_ = *δ*^2^H_Captive_  100% hits in the Discriminant Analysis  *P. reticulatus*:  *δ*^13^C_wild-Vietnam_<*δ*^13^C_wild-Indonesia=_*δ*^13^C_captive-Vietnam_  *δ*^2^H_wild-Vietnam_< *δ*^2^H_wild-Indonesia =_ *δ*^2^H_captive-Vietnam_  *δ*^15^N_wild-Vietnam=_ *δ*^15^N_wild-Indonesia =_ *δ*^15^N_captive-Vietnam_ | (Natusch et al., 2017) |
|  | *Trachemys scripta elegans* | *δ*^13^C e *δ*^15^N | Carapace | Australia | *δ*^15^N_wild_ < *δ*^15^N_captive_  Assignment test: minimum accuracy of 96% | (Hill et al., 2020) |
| **BIRD** | *Carduelis carduelis* | *δ*^2^H | Feather | England | *δ*^2^H_C.c. major_ < *δ*^2^H_C.c. brittanica_ = *δ*^2^H_Captive_ | (Kelly, Thompson & Newton, 2008) |
|  | *Colinus virginianus* | *δ*^13^C, *δ*^15^N, *δ*^34^S, *δ*^2^H | Feather | United States | 100% hits in the Discriminant Analysis (wild *vs.* captive); 99% hits in the Discriminant Analysis (different farms)  Isotopes used: *δ*^13^C, *δ*^15^N, *δ*^34^S, | (Castelli & Reed, 2017) |
|  | *Psittacus erithacus* | *δ*^13^C*, δ*^15^N, *δ*^2^H | Feather | South Africa | *δ*^13^C_wild_ < *δ*^13^C_captive_  *δ*^2^H_wild_ < *δ*^2^H_captive_ | (Alexander et al., 2019) |
|  | *Emberiza hortulana* | *δ*^2^H | Feather | France | *δ*^2^H_wild_ > δ^2^H_captive_ | (Jiguet, Kardynal & Hobson, 2019) |
|  | *Cacatua sulphurea, Cacatua sp.* | *δ*^13^C, *δ*^15^N | Feather | China | *δ*^13^C_wild_ < *δ*^13^C_captive;_ *δ*^15^N_wild_ > *δ*^15^N_captive_  LDA_wild vs. captive_: Accuracy = 0.91 | (Andersson et al., 2021) |
| **MAMMAL** | *Mustela vison* | *δ*^13^C | Teeth; claw | Denmark | *δ*^13^C_wild_ < *δ*^13^C_captive_  95.9% correct classification to the supposed origin group | (Hammershøj et al., 2005) |
|  | *Canis lupus* | *δ*^13^C, *δ*^15^N | Hair; bone | USA | *δ*^13^C_wild_ < *δ*^13^C_captive;_ *δ*^15^N_wild_ = *δ*^15^N_captive_  (Based on no overlaps between groups) | (Kays & Feranec, 2011) |
|  | *Tachyglossus aculeatus* | *δ*^13^C, *δ*^15^N | Quills | Australia | *δ*^13^C_wild_ < *δ*^13^C_captive;_ *δ*^15^N_wild_ < *δ*^15^N_captive_  91.31% correct classification | (Brandis et al., 2018) |
|  | *Panthera leo* | *δ*^13^C e *δ*^15^N | Hair | Australia | *δ*^15^N_wild_ > *δ*^15^N_captive_  Predictive model_wild vs. captive_: Accuracy = 0.7 | (Hutchinson & Roberts, 2020) |

^*Not included in the database (.xlsx file) because isotopic data were not available^
