# Supplementary material for: Differentiating wild from captive animals: an isotopic approach
Source: PeerJ. 2023 Nov 24;11:e16460. doi: 10.7717/peerj.16460 (PMC10680447; doi:10.7717/peerj.16460)
Supplement: Supplemental Information 3 — In the “Summary proposal,” DTDF means diet-tissue discrimination factors. In “Summary results,” the equal sign (=) indicates the absence of significant differences in inferential tests. [file peerj-11-16460-s003.docx]

**Table S3.** Summary of studies measuring stable isotopes in wild and captive animals organized by taxon group (fish, amphibian, reptile, bird, and mammal). In the “Summary proposal,” DTDF means diet-tissue discrimination factors. In “Summary results,” the equal sign (=) indicates the absence of significant differences in inferential tests.

|  | **TAXON (SPECIE)** | **ISOTOPES ANALYZED** | **TISSUE** | **LOCAL (COUNTRY)** | **SUMMARY PROPOSAL:** | **SUMMARY RESULT** | **REFERENCE** |
| --- | --- | --- | --- | --- | --- | --- | --- |
| **FISH** | *Thunnus thynnus* | *δ*^13^C, *δ*^15^N | Muscle and liver | Italy | To assess the changes occurring during farming, investigate the sources of nutrition for *T. thynmus*. | *δ*^13^C_wild_ = *δ*^13^C_captive_  *δ*^15^N_wild_ < *δ*^15^N_captive_ | (Vizzini, Tramati & Mazzola, 2010)^*^ |
|  | *Salmo trutta;* | *δ*^13^C, *δ*^34^S | Scale | Poland | To analyze the relationship between sulfur in sulfate dissolved in water and in fish scales | *δ*^13^C_wild-river_ < *δ*^13^C_captive;_  *δ*^34^S_wild-river_ < *δ*^34^S_captive_ | (Trembaczowski & Niezgoda, 2011) |
|  | *Belone belone; Boops boops* | *δ*^13^C, *δ*^15^N | Muscle | Croatia | To assess the presence, concentrations, origin, and fate of targeted metals and the effects farming has on wild fish. | *δ*^13^C_wild_ = *δ*^13^C_captive_  *B. belone: δ*^15^N_wild_ = *δ*^15^N_captive_  *B. boops: δ*^15^N_wild_ < *δ*^15^N_captive_ | (Fernandez-Jover et al., 2020)^*^ |
| **REPTILE** | *Sphenodon punctatus* | *δ*^13^C | Blood (RBC) | New Zeland | To make inferences about marine content in the diet of *S. punctatus* in response to seasonality and life story. | *δ*^13^C_wild_ = *δ*^13^C_captive_ | (Cree et al., 1999) |
|  | *Alligator mississippiensis* | *δ*^18^O | Bone | USA | To analyze the inter and intra-bone variability of *δ*^18^O according to temperature regularity. | *δ*^18^O_wild_ < *δ*^18^O_captive_ | (Stoskopf, Barrick & Showers, 2001) |
|  | *Bothrops atrox* | *δ*^13^C, *δ*^15^N | Blood and scale | Brazil | To analyze the influence of different landscapes on the diet of *B. atrox* | Blood:  *δ*^13^C_wild_ = *δ*^13^C_captive_  *δ*^15^N_wild_ = *δ*^15^N_captive_  Scale:  *δ*^13^C_wild_forest_ < *δ*^13^C_captive_  *δ*^15^N_wild_forest_ > *δ*^15^N_captive_ | (Martinez, 2016) |
| **BIRD** | *Cerorhinca monocerata* | *δ*^13^C, *δ*^15^N | Blood and feather | USA | To examine the effects of growth and nutritional status on stable isotope signatures in *C. monocerata* tissues. | *δ*^13^C_wild_ < *δ*^13^C_captive;_  *δ*^15^N_wild_ < *δ*^15^N_captive_ | (Sears, Hatch & O’Brien, 2009)^*^ |
|  | *Fratercula arctica; Uria aalge* | *δ*^13^C, *δ*^15^N | Blood (RBC and plasma) | Canada | To estimate the DTDFs for captive *F. artica* and *U. aalge* and to reconstruct the diet of wild breeding individuals of the same species | *δ*^13^C_wild_ < *δ*^13^C_captive;_  *δ*^15^N_wild_ < *δ*^15^N_captive_  (RBC and plasma, both species) | (Jenkins et al., 2020) |
| **MAMMAL** | *Mustela vison* | *δ*^13^C | Claw and teeth | Denmark | To conduct a diet-change experiment to verify if the SIA could identify farm-scaped minks (*M. vison*). | *δ*^13^C_wild_ = *δ*^13^C_captive_  (Claw and teeth) | (Hammershøj, Asferg & Kristensen, 2004) |
|  | *Hydrochoerus hydrochaeris* | *δ*^13^C, *δ*^15^N | Blood, claw, hair, and muscle | Brazil | To analyze the diet composition of *H. hydrochaeris* and the reliability of using stable isotopes as a proxy | Blood, claw, and hair:  *δ*^13^C_wild_ = *δ*^13^C_captive_  *δ*^15^N_wild_ < *δ*^15^N_captive_  Muscle:  *δ*^13^C_wild_ > *δ*^13^C_captive_  *δ*^15^N_wild_ = *δ*^15^N_captive_ | (Navarro, 2009) |
|  | *Phoca vitulina* | *δ*^13^C, *δ*^15^N | Blood (serum) | USA | To determine the trophic level and DTDFs of different tissues and groups of harbor seals | *δ*^13^C_wild_ > *δ*^13^C_captive;_  *δ*^15^N_wild_ > *δ*^15^N_captive_ | (Germain et al., 2012) |
|  | *Loxodonta africana* | *δ*^13^C, *δ*^15^N | Hair | South Africa | To compare the patterns of seasonal dietary variability across individuals of *L. Africana.* | *δ*^13^C_wild_ < *δ*^13^C_captive;_  *δ*^15^N_wild_ > *δ*^15^N_captive_ | (Codron et al., 2013) |
|  | *Otaria flavescens* | *δ*^13^C, *δ*^15^N | Blood (RBC and serum) | Uruguay; Spain | To estimate DTDF for females and pups *O. flavescens* in the wild and captive. | *δ*^13^C_wild_ > *δ*^13^C_captive;_  *δ*^15^N_wild_ > *δ*^15^N_captive_ | (Drago et al., 2015)^*^ |
|  | *Otaria flavescens* | *δ*^13^C, *δ*^15^N | Vibrissae | Argentina; Spain | To analyze the fluctuation in stable isotope values along the vibrissae from wild adult breeding *O. flavescens* | *δ*^13^C_wild_ > *δ*^13^C_captive;_  *δ*^15^N_wild_ > *δ*^15^N_captive_ | (Cardona et al., 2017) |

^*We could not access or infer the original database. The inferences were based on the mean and standard deviation.^
