# Supplementary material for: Differentiating wild from captive animals: an isotopic approach
Source: PeerJ. 2023 Nov 24;11:e16460. doi: 10.7717/peerj.16460 (PMC10680447; doi:10.7717/peerj.16460)
Supplement: Supplemental Information 4 — LC = “Least Concern”; NT = “Near Threatened”; VU = “Vulnerable”; EN = “Endangered”; CR = “Critically Endangered”; RE = “Regionally Extinct”, NE = “Not Evaluated” and DD = “Data Deficient”. [file peerj-11-16460-s004.docx]

**Table S4.** Global status of the species used in “wild *vs.* captive” studies on the IUCN red list, CITES appendices, and summary of the captive information available in each paper. LC = “Least Concern”; NT = “Near Threatened”; VU = “Vulnerable”; EN = “Endangered”; CR = “Critically Endangered”; RE = “Regionally Extinct”, NE = “Not Evaluated” and DD = “Data Deficient”.

| **REFERENCE** | **TAXON** | **IUCN STATUS** | **CITES** | **CAPTIVE INFORMATION** |
| --- | --- | --- | --- | --- |
| (Dempson & Power, 2004) | *Salmo salar* | LC | No | Samples from a strain of *S. salar* farmed exclusively in sea-cage culture. The commercial food supplied was also analyzed. |
| (Rojas et al., 2007) | *Sparus aurata* | LC | No | Samples of *S. aurata* from local producers in four Mediterranean countries. Captive conditions are unknown. However, interviews with fish farmers indicated that farmers in the same country tend to purchase feeds from a unique national provider. |
| (Bell et al., 2007) | *Dicentrarchus labrax* | LC | No | Samples of *D. labrax* from a research laboratory in Scotland and a farm company in Greece. No further information about the captive was provided. However, the authors mention that sea bass used to be fed a commercial Atlantic cod diet, with lower lipid content than bass diets produced in southern Europe. |
| (Molkentin et al., 2007) | *Salmo salar* | LC | No | Samples of conventionally and organically reared *S. salar* were purchased. The captive conditions are unknown. However, there are some standards for organic aquaculture, such as all feeding stuff shall be of a certified organic origin; fish meal or oil shall come from the same geographical region and shall be obtained from by-products of wild-caught fish for human consumption; the use of synthetic feed additives is not allowed. |
| (Serrano, Blanes, & Orero, 2007) | *Sparus aurata* | LC | No | Samples of sea-cage-farmed *S. aurata* were purchased from local commercial markets. No further information about captive was provided, but the authors used morphology and lipid content to confirm the fish origin (wild or captivity) |
| (Busetto et al., 2008) | *Psetta maxima* | LC | No | Samples of *P. maxima* were collected at the wholesale fish market and by local retailers. No further information about the captive was provided. |
| (Anderson, Hobbie, & Smith, 2010) | *Oncorhynchus tshawytscha*  *Oncorhynchus kisutch;*  *Salmo salar* | NE  NE  LC | No | Samples of *O. tshawytscha, O. kisutch,* and *S. salar* from five aquaculture facilities. No further information about the captive was provided. |
| (Fasolato et al., 2010) | *Dicentrarchus labrax* | LC | No | Samples of *D. labrax* from three intensive farms in Italy and Greece. No further information about the captive was provided. However, the authors mention that the European sea bass can be farmed extensively in brackish lagoons and intensively in floating cages or in-shore ponds, employing high nutritional feed. |
| (Sant’Ana, Ducatti, & Ramires, 2010) | *Pseudoplatystoma fasciatum* | NE | No | Samples from *P. fasciatum* were obtained from two local fish farms in the dry and rainy seasons, where they were fed a commercial diet and small fish. |
| Schröder and Leaniz, 2011 | *Salmo salar*  *Oncorhynchus mykiss* | LC  NE | No | Samples of *S. salar* and *O. mykiss* were collected at two local fish farms. No further information about the captive was provided. |
| (Trembaczowski & Niezgoda, 2011) | *Salmo trutta*  *Oncorhynchus mykiss* | LC | No | Samples of *O. mykiss* were caught in commercial ‘put&take’ fishery supplied directly from commercial pond farms. Fishes used to stay no longer than a month in such ponds.  Samples of *S. trutta* from a hatchery from where almost all trouts and graylings were used to stock rivers in the study region. |
| (Molkentin et al., 2015) | *Salmo salar;*  *Salmo trutta* | LC  LC | No | Samples of organically and conventionally farmed salmons were purchased from retail stores and wholesale (*S. salar*) or directly from fish farms (*S. trutta*). The commercial food supplied was also analyzed. No further information about the captive was provided. However, guidelines for organic farming require at least 40% of animal content for carnivorous species. In conventional farms, more than 60% of vegetable ingredients are allowed. |
| (Chaguri et al., 2017) | *Argyrosomus regius* | LC | No | Samples from *A. regius* cultivated in earth ponds from a local aquaculture facility. No further information about the captive was provided. |
| (Farabegoli et al., 2018) | *Dicentrarchus labrax* | LC | No | Samples of *D. labrax* from intensive (up to 30 kg m^-3^), semi-intensive (up to 1 kg m^-3^), and extensive (up to 0.0025 kg m-3) rearing farms. The intensive farms were equipped with either floating or submersible cages, the semi-intensive with earthen tanks, and the extensive farm was based in valliculture. |
| (Wang et al., 2018) | *Salmo salar* | LC | No | Samples of *S. salar* came from a known organic producer or were purchased at the supermarket labeled as organically or conventionally reared. The authors assume that farmed salmon obtained from supermarkets were raised according to the EU regulation: organic salmon are fed at least 40% marine originated diet, and conventional are fed less than 22% of marine origin since 2015. |
| (Gopi et al., 2019) | *Lates calcarifer* | LC | No | Samples of *L. calcarifer* came from the wholesale market through collaboration with industry and research partners. The samples were randomly collected from different ponds at each farm. No further information about the captive was provided. However, the study mentions that *L. calcarifer* has been farmed in brackish water, freshwater, and  marine conditions. Pond or net-cage culture is the preferred method of cultivation. |
| (Pereira et al., 2019) | *Arapaima spp.* | DD | II | Samples from *Arapaima spp.* farms or markets. No further information about the captive was provided. |
| (Vasconi et al., 2019) | *Anguilla anguilla* | CR | II | Samples from *A. anguilla* farm or purchased on a retail market labeled as from Netherlands, Denmark, or Italy. The authors point out two main rearing systems of eels in Europe: extensive rearing in ponds or vallicoltura (practiced by Italian farmers) or intensive rearing in which eels are kept at their optimum temperature and fed with extruded dry feed several times a day (practiced by Dutch and Danish farmers). |
| (Nabaes Jodar, Cussac, & Becker, 2020) | *Oncorhynchus mykiss* | NE | No | Samples from three *O. mykiss* farms in the same region of wild ones (Alicurá reservoir). No further information about the captive was provided. |
| (Liu et al., 2020) | *Cyprinus carpio*  *Ctenopharyngodon idella;*  *Hypophthalmichthys molitrix;*  *Mylopharyngodon piceus* | VU  LC  NT  LC | No | Samples of lake-farmed and pond-farmed carp. Lake-farmed carp were collected from 4 large fish enclosures (each>10 km2) and fed under protocols that comply with Organic Aquaculture Certification Standards. Pond-farmed carp were collected from 20 ponds. Water was piped directly from a regional lake into these large artificial ponds (depth>3 m) and exchanged every 15 days. The density of carp in pond-farmed systems was higher than lake-farmed carp by at least 20 fish per m^2^, and the fishes were intensively fed (> 5 kg per m^2^ per day) using plant and animal-based proteins. |
| (Dittrich, Struck, & Rödel, 2017) | *Hoplobatrachus rugulosus;*  *Fejervarya cancrivora; Limnonectes macrodon* | LC  LC  LC | No | Samples from three species of deep-frozen frog legs from Vietnam and Indonesia were bought in supermarkets in Germany. Captive conditions are unknown. However, according to the package labels and the authors’ conclusions, Vietnamese samples are from frog farms, and Indonesian samples are from wild or free-ranging farming. |
| (van Schingen et al., 2016) | *Shinisaurus crocodilurus* | EN | I | Samples from *S. crocodilrus* born in captivity. Adults are kept in groups of three to four individuals in outdoor enclosures of about 2–7 m^2^, while juveniles are kept in small groups or pairs within plastic boxes inside the station during the first months. Animals are fed once or twice a week, mainly with beetle larvae and sometimes earthworms and crickets, while juveniles are fed more frequently. |
| (Natusch et al., 2017) | *Python reticulatus;*  *Python bivittatus* | LC  VU | II | Samples from *P. reticulatus* reared on a diet of wild-caught rats (*Rattus argentiventer*) at a commercial breeding facility.  Samples from *P. bivittatus* born on a python farm and raised in a dietary experiment for 13 months. The experiment included wild rats or sausages made from reconstituted waste protein, the predominant items on captive *P. bivittatus* diet in Viet Nam. |
| (Hill et al., 2020) | *Trachemys scripta* | LC | No | Samples from *T. scripta* seized by state wildlife compliance agencies. Specimens were classified as “wild” or “captive” based on the assumed environmental history. Captive conditions are unknown. |
| (A. Kelly, Thompson, & Newton, 2008) | *Carduelis carduelis* | LC | III | Samples from captive-bred *S. canaria*. No further information about the captive was provided. |
| (Castelli & Reed, 2017) | *Colinus virginianus* | NT | I | Samples from hunting farms of *C. virginianus*. The commercial food supplied was also analyzed. No further information about captive was provided; however, the standard management of *C. virginianus* includes getting the eggs from dealers, moving young chicks into flight pens with a density of 2ft^2^, and high-protein food.^1^ |
| (Alexander et al., 2019) | *Psittacus erithacus* | EN | I | Samples from *P. erithacus* kept in captivity for at least one year. No further information about the captive was provided. |
| (Jiguet, Kardynal, & Hobson, 2019) | *Emberiza hortulana* | LC | III | Samples from *E. hortulana* seized by the police. Captive conditions are unknown; however, the authors selected individuals with plumage dysfunctions characteristics of long-term captivity since there is no captive breeding of the species. |
| (Andersson et al., 2021) | *Cacatua sulphurea* | CR | I | Samples from five species of the genus *Cacatua* kept in captivity for at least one year and completed at least one molt during that time. Birds were held by research centers, private owners, pet shops, and zoos. The commercial food supplied was also analyzed. |
| (Mette Hammershøj et al., 2005) | *Mustela vison* | LC | No | Samples from free-ranging *M. vison* to identify individuals’ origin. Captive conditions are unknown. However, the authors assume that the food used in most farm mink in Denmark has a high marine fish content and is acquired from a few feed mills. |
| (Kays & Feranec, 2011) | *Canis lupus* | LC | I; II | Samples from captive *C. latrans* fed scrap beef and kibbled dog food. No further information about the captive was provided. |
| (Brandis et al., 2018) | *Tachyglossus aculeatus* | LC | No | Samples from *T. aculeatus* kept at the Zoo for between 22 months and 20 years since they are challenging to breed in captivity. All captive echidnas were fed the same diet. |
| (Hutchinson & Roberts, 2020) | *Panthera leo* | VU | I; II | Samples from South African *P. leo* provided by taxidermists. Captive conditions are unknown. |

1. ^https://extension.psu.edu/bobwhite-quail-production^
