# Supplementary material for: Differentiating wild from captive animals: an isotopic approach
Source: PeerJ. 2023 Nov 24;11:e16460. doi: 10.7717/peerj.16460 (PMC10680447; doi:10.7717/peerj.16460)
Supplement: Supplemental Information 5 — We could access or infer the original data from 10 of the 14 publications. [file peerj-11-16460-s005.docx]

**Table S5**. Comparison of the mean isotopic ratios in captive and wild animals of the publication that measured but did not testes for differences. We could access or infer the original data from 10 of the 14 publications.

| **REFERENCE** | **SPECIE** | **TISSUE** | **ISOTOPE** | **TEST** | **RESULT** |
| --- | --- | --- | --- | --- | --- |
| Trembaczowski & Niezgoda, 2011 | *Salmo trutta* | Scale | *δ*^13^C | Wilcox test | *W* = 468; *p* < 0.001 |
|  |  |  | *δ*^34^S | Wilcox test | *W* = 380; *p* < 0.001 |
| Cree et al., 1999 | *Sphenodon punctatus* | Red blood cells | *δ*^13^C | Wilcox test | *W* = 517; *p* = 0.191 |
| Stoskopf, Barrick & Showers, 2001 | *Alligator mississippiensis* | Bone | *δ*^18^O | Wilcox test | *W* = 5767; *p* < 0.001 |
| Martinez, 2016 | *Bothrops atrox* | Scale | *δ*^13^C | Kruskal-Wallis | *H_3_* = 7.304; *p* = 0.062 |
|  |  |  | *δ*^15^N | One way ANOVA | *F*_3,36_ = 19.5; *p* < 0.001 |
|  |  | Blood | *δ*^13^C | One way ANOVA | *F*_3,28_ = 0.412; *p* = 0.745 |
|  |  |  | *δ*^15^N | Kruskal-Wallis | *H_3_* = 3.150; *p* = 0.368 |
| Jenkins et al., 2020 | *Fratercula arctica* | Plasma | *δ*^13^C | Wilcox test | *W* = 281; *p* < 0.001 |
|  |  |  | *δ*^15^N | Wilcox test | *W* = 304; *p* < 0.001 |
|  |  | Red blood cells | *δ*^13^C | Student’s t-test | *t*_33_ = 34.271; *p* < 0.001 |
|  |  |  | *δ*^15^N | Student’s t-test | *t*_33_ = 57.588; *p* < 0.001 |
|  | *Uria aalge* | Plasma | *δ*^13^C | Wilcox test | *W* = 328; *p* < 0.001 |
|  |  |  | *δ*^15^N | Wilcox test | *W* = 380; *p* < 0.001 |
|  |  | Red blood cells | *δ*^13^C | Student’s t-test | *t*_37_ = 36.614; *p* < 0.001 |
|  |  |  | *δ*^15^N | Welch t-test | *t*_26.04_ = 35.57; *p* < 0.001 |
| Hammershøj, Asferg & Kristensen, 2004 | *Mustela vison* | Claw | *δ*^13^C | Welch t-test | *t*_7.028_ = 1.040; *p* = 0.332 |
|  |  | Teeth | *δ*^13^C | Wilcox test | *W* = 24; *p* = 0.730 |
| Navarro, 2009 | *Hydrochoerus hydrochaeris* | Blood | *δ*^13^C | Wilcox test | *W* = 255; *p* = 0.855 |
|  |  |  | *δ*^15^N | Wilcox test | *W* = 342; *p* < 0.030 |
|  |  | Claw | *δ*^13^C | Student’s t-test | *t*_48_ = 0.381; *p* = 0.704 |
|  |  |  | *δ*^15^N | Wilcox test | *W* = 433; *p* < 0.010 |
|  |  | Hair | *δ*^13^C | Student’s t-test | *t*_48_ = 0.501; *p* = 0.618 |
|  |  |  | *δ*^15^N | Wilcox test | *W* = 420; *p* = 0.017 |
|  |  | Muscle | *δ*^13^C | Wilcox test | *W* = 54.5; *p* < 0.001 |
|  |  |  | *δ*^15^N | Wilcox test | *W* = 300; *p* = 0.227 |
| Germain et al., 2012 | *Phoca vitulina* | Serum | *δ*^13^C | Student’s t-test | *t*_109_ = -7.891; *p* < 0.001 |
|  |  |  | *δ*^15^N | Wilcox test | *W* = 228.5; *p* < 0.001 |
| Codron et al., 2013 | *Loxodonta africana* | Hair | *δ*^13^C | Linear mixed model | AIC = 1675.0; χ < 0.001 |
|  |  |  | *δ*^15^N | Linear mixed model | AIC = 1815.8; χ < 0.001 |
| Cardona et al., 2017 | *Otaria flavescens* | Vibrissae | *δ*^13^C | Linear mixed model | AIC = 641.61; χ < 0.001 |
|  |  |  | *δ*^15^N | Linear mixed model | AIC = 876.58; χ < 0.001 |
