# Supplementary figures and images for: Differentiating wild from captive animals: an isotopic approach
Source: PeerJ. 2023 Nov 24;11:e16460. doi: 10.7717/peerj.16460 (PMC10680447; doi:10.7717/peerj.16460)

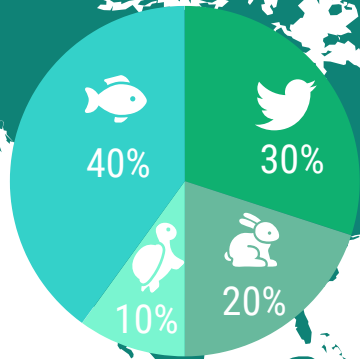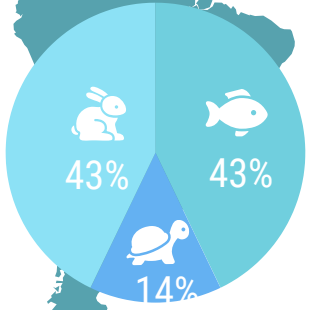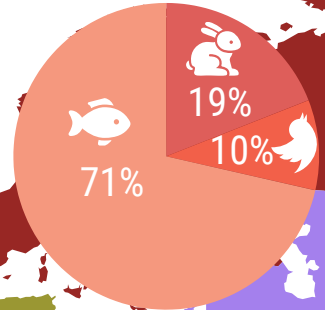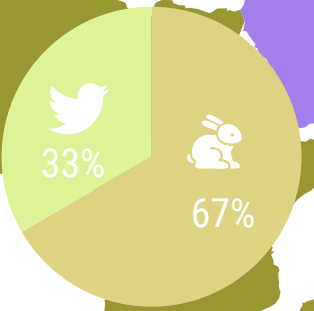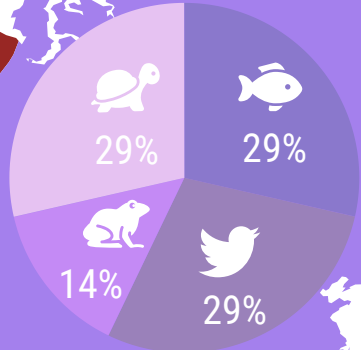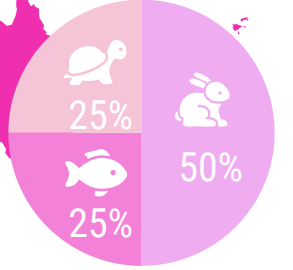

Supplement: Supplemental Information 6 [file peerj-11-16460-s006.pdf]

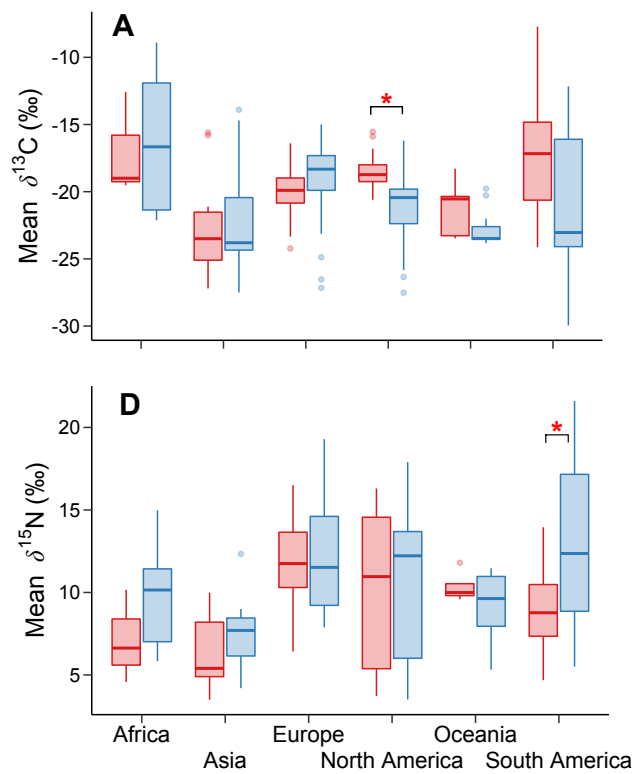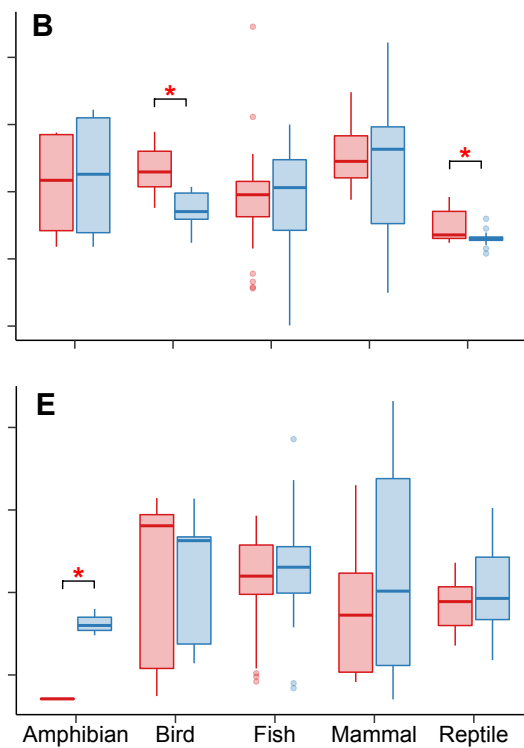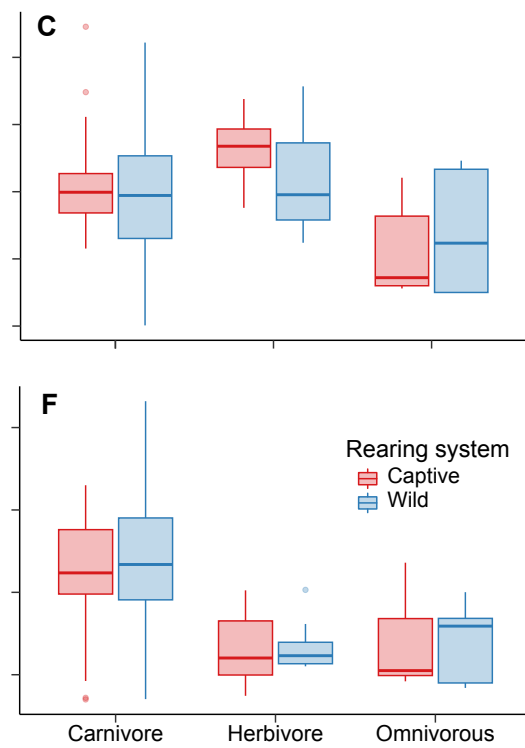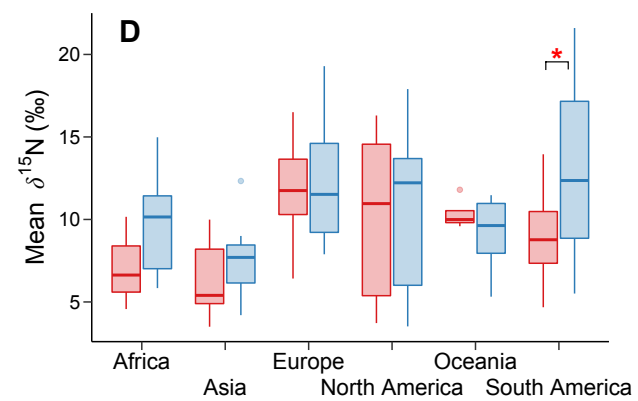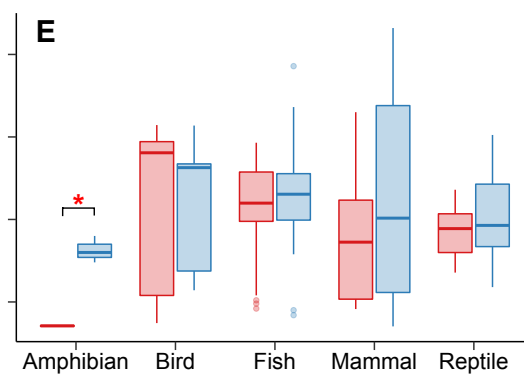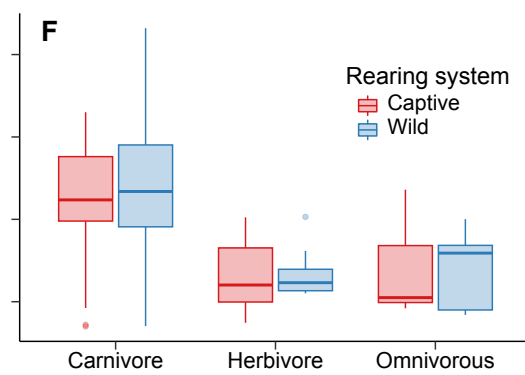

Supplement: Supplemental Information 7 — The box represents the middle 50% of the data, and whiskers extend to the highest and lowest values within 1.5 times the interquartile range. Asterisks indicate significant differences (p < 0.05). [file peerj-11-16460-s007.pdf]

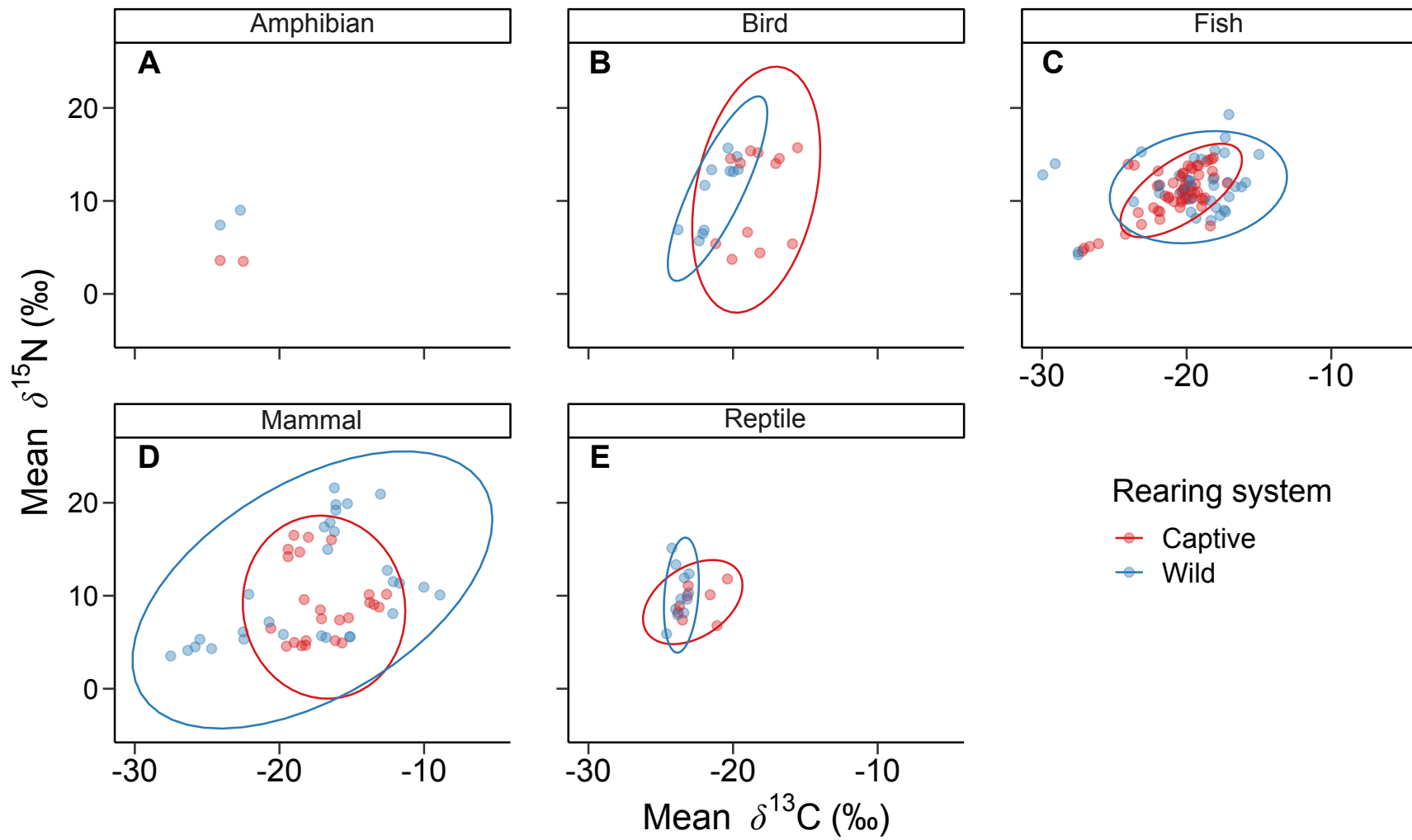

Supplement: Supplemental Information 8 [file peerj-11-16460-s008.pdf]
